# Lymphoid aggregates in the bone marrow biopsies of patients with myelodysplastic syndromes – A potential prognostic marker?

**DOI:** 10.3389/fonc.2022.988998

**Published:** 2023-01-26

**Authors:** Reut Book, Jonathan Ben-Ezra, Chen Glait Santar, Sigi Kay, Galia Stemer, Howard S. Oster, Moshe Mittelman

**Affiliations:** ^1^ Department of Medicine A, Tel Aviv Sourasky Medical Center, Tel Aviv, Israel; ^2^ Department of Pathology, Tel Aviv Sourasky Medical Center, Tel Aviv, Israel; ^3^ Sackler Faculty of Medicine, Tel Aviv University, Tel Aviv, Israel; ^4^ Hematology Laboratory, Tel Aviv Sourasky Medical Center, Tel Aviv, Israel; ^5^ Hematology Institute, Galilee Medical Center, Nahariya, Israel; ^6^ Faculty of Medicine, Bar Ilan University, Safed, Israel; ^7^ Department of Hematology, Tel Aviv Sourasky Medical Center, Tel Aviv, Israel

**Keywords:** lymphoid aggregates, myelodysplastic syndromes, bone marrow biopsy, prognosis, survival

## Abstract

**Background:**

Lymphoid aggregates (LA) are occasionally seen in bone marrow biopsies (BMB) of myelodysplastic syndromes (MDS) patients. Our aim was to evaluate their incidence and association with prognosis.

**Methods:**

We compared BMB reports of MDS patients treated at the Tel Aviv Sourasky Medical Center (2011-2018), and controls (2015-2017, normal BMB), and examined the charts of the MDS patients (LA+ and LA-). Categorical, normally and non-normally distributed continuous variables were compared using Fisher’s exact, independent t and Mann-Whitney tests respectively. Adjusted [age, gender, lymphocytes, white blood cells (WBC) and diabetes mellitus (DM)] Cox proportional hazard model examined survival at 12 and 24 months.

**Results:**

MDS patients (N=140) were older than controls (N=38; 74.1 vs 69.2 years, p=0.005); 34 MDS (24.3%) and 5 controls (13.2%) had LA+ (P=0.141). CD20/CD3 staining suggested LA polyclonality. MDS/LA+ (vs MDS/LA-) patients were younger, with a trend (not statistically significant) towards poor prognostic parameters: lower Hb, WBC, and platelets, higher LDH, BM cellularity, and IPSS-R score. The incidence of cardiovascular disease was similar, but MDS/LA+ had twice the incidence of DM (38.2% vs 19.0%, p=0.022). Similar trend for cancer (26.5% vs 14.3%, p=0.102). Twelve-month survival: 24/34 (70.6%) MDS/LA+; 88/106 (83.0%) MDS/LA- (p=0.140). This trend, seen in Kaplan-Meier curves, disappeared at 24 months. The hazard ratio for LA was 2.283 (p=0.055) for 12 months.

**Conclusion:**

These preliminary data suggest LA are relatively common (24%) in MDS BMB, and might indicate poor prognosis. This may reflect involvement of the immune system in MDS. Future studies will examine larger groups, to clarify the incidence, significance and the pathophysiology.

## Introduction

Myelodysplastic syndromes (MDS) are clonal stem cell disorders characterized by ineffective hematopoiesis, cytopenia and a likelihood of leukemic transformation (1, 2). Bone marrow (BM) examination, aspiration and/or biopsy, remains the gold standard for diagnosis with the typical dysplastic features in one or more lineages (3–5). The BM blast percentage serves for both assisting in diagnosis and predicting prognosis. Other BM findings that are occasionally reported in MDS patients, such as fibrosis (6), and clusters of abnormal localization of immature (myeloid) precursors (ALIP) have been proposed as prognostic factors (7–9), but have not been introduced into the prognostic models (6, 10–13). Often, BM biopsy (BMB) report includes an estimate of cellularity, however, this has not become an important diagnostic or prognostic tool either (3, 4).

Clusters of lymphoid aggregates (LA) are occasionally observed in BMB of patients with non-lymphoproliferative disorders. We have noticed that, in addition to the known dysplastic features, LA are commonly reported as present in BMB of patients diagnosed with myelodysplastic syndromes (MDS). While this finding is probably not specific and is not a part of the MDS diagnostic or prognostic tools, it raises several questions, including the incidence of this finding, as well as its role in the pathogenesis and possibly disease prognosis.

In many non-malignant, hematological or other conditions, including systemic autoimmune diseases and infectious disorders, such as HIV, hepatitis B or C, bacterial, mycobacterial and fungal infections, LA can be seen in BMB (14–16). These LA are often polyclonal and are believed to represent an immune reaction and an inflammatory condition that is part of the disease pathophysiology (14, 15). Interestingly, an increased incidence of benign aggregates in lymphoma patients who have been treated with rituximab has also been reported (14).

Occasionally in an otherwise “normal” BM, LA can be the harbinger of a lymphoproliferative disorder that will be diagnosed later (14). The predominance of B cells within the aggregates, the presence of a core of B cells surrounded by T cells (except in germinal center formation), cytologic atypia, paratrabecular location, infiltrative edges, and large LA that increase in size in deeper sections, are all features that should raise suspicion of BM involvement by a lymphoproliferative disorder (14, 17). As expected, in such disorders (e.g. lymphomas and CLL), in addition to possible lymphoid infiltration, the BMB sample can include LA, which are often monoclonal malignant (18).

The presence of LA in MDS BMB has received little attention so far. In the few publications, the incidence ranges from 2.5 to 25% of patients (15, 16, 19–22). In the series of Magalhães et al. (20), 51 out of 206 MDS patients had LA in the BMB. MDS patients with LA did not differ from those without LA in age, gender distribution and other common parameters. Although MDS patients with LA had lower Hb and increased reticulin fibers, and were more associated with higher-risk disease, these failed to reach statistical significance and the prognosis related to leukemic progression and overall survival was similar in both groups. Other studies evaluated the correlation between LA and several other parameters, including MCV, monocyte or eosinophil counts, BM cellularity, and overall survival, but none of these reports had an impact on the diagnostic or prognostic paradigm (22–24).

We report here a single-institute small-scale analysis, studying the characteristics, incidence and potential prognostic role of the presence of LA in the BMB reports of an MDS patient cohort.

## Patients and methods

### Patients

We reviewed the medical records of patients diagnosed, followed or treated at the Tel-Aviv Sourasky Medical Center, from January 2011 through December 2018. For this retrospective analysis patients had to fulfill the following criteria: MDS Diagnosis (1, 25) with an available baseline BM biopsy report at diagnosis, BM biopsy had been interpreted by a single hemato-pathologist (JBE). Because lymphoid aggregates are not normally part of the routine BM assessment for MDS, they may be overlooked. The requirement of a single pathologist was put in place because our hematopathologist is particularly minded to LA, and the data are more consistent and objective. Patient charts were reviewed and data were examined from the time of diagnosis, before any treatment was given.

Patients were excluded from the analysis if MDS diagnosis was only suspected but not yet proven, if a baseline BMB was not available or if only an aspirate had been performed. Patients were also excluded if they had another concurrent hematologic or neoplastic disease.

The controls were consecutive patients undergoing BMB as a part of a workup for unexplained anemia (2015-2017), who had an available BMB report from the time of the evaluation (the same hemato-pathologist, JBE) that had been interpreted as normal or non-diagnostic. Control patients were excluded if they had any current hematological or oncologic disease diagnosed or present in the BM or elsewhere in the body.

### Methods

First, we compared the MDS patient population with the non-MDS controls with a special focus on the presence or absence of LA, as well as other demographic parameters.

The main analysis was a comparison between the two MDS patient subpopulations: MDS patients whose BMB was reported to include clusters of LA (LA+), and those MDS patients in whom LA were not reported (LA-). For the comparison, we used a categorical parameter – presence or absence of LA in the baseline BMB report.

The two MDS patient subpopulations were compared with regard to several baseline parameters from chart reviews at the time of diagnosis: demographic characteristics, comorbidities, medications, lab values – especially hemoglobin (Hb), mean corpuscular volume (MCV), white blood cell (WBC) count, absolute neutrophil count (ANC), lymphocyte count, lactate dehydrogenase (LDH) – BM cellularity and percentage of blast cells, international prognostic scoring system (IPSS) and IPSS-R (IPSS revised) scores. We also assessed the BM lymphoid cell characteristics using the immunohistochemistry staining reports. This staining had been performed in the clinical pathology lab of our medical center, using the Benchmark ULTRA (Roche Diagnostics) automated device and protocol.

The main outcome was survival, specifically, the number/percentage of patients alive in each group at 12 and at 24 months. We also looked at the flow cytometry pattern of the BM aspirates in the subset of the patients where the data were available. We chose to focus on the CD34+ cells (stem cells). We also examined the percentage of lymphocytes in the BM, as well as the percentage of BM T cells and B cells (CD7 and CD19, respectively) and the percentage of lymphocytes that were T or B cells.

### Statistical analysis

Comparisons were made between MDS patients and non-MDS patients and between MDS patients with and without lymphoid aggregates as described in the text. Categorical variables are summarized with count & percentages and compared between groups using Fisher’s exact test. Continuous variables are summarized with means ± standard deviation if distribution is approximately normal or with medians [interquartile range, IQR] if normality cannot be assumed, and comparisons between groups were performed using independent t-test or Mann Whitney test respectively.

A Kaplan Meir model was applied to compare the survival at 12 and 24 months.

A Cox proportional hazard model was applied to evaluate the survival adjusted for age, gender, lymphocytes, WBC and diabetes at 12 and 24 months. P values <0.05 were considered as statistically significant. Analyses were carried out using SPSS 27.0.

## Results

In total, 178 patients, 140 with myelodysplastic syndromes (MDS), and 38 controls, met the criteria, and entered the study (**Table 1**). The MDS patients were found to be older (mean 74.1 years) than the controls (69.2 years, p=0.005). The gender distribution within the two analyzed groups was similar: male/female ratio 58.6/41.4% and 57.9/42.1% (P=0.940), in the MDS and controls, respectively.

**Table 1:** Patient characteristics.

N	MDS	Controls	Total	P
	140	38	178	
Age years (mean ± SD)	74.1 ± 10.8	69.2 ± 9.9	73.1 ± 10.8	0.005
Male N (%)	82 (58.6%)	22 (57.9%)	104 (58.4%)	0.940
Female N (%)	58 (41.4%)	16 (42.1%)	74 (41.6%)	
LA+ N (%)	(34) 24.3%	(5) 13.2%	(39) 21.9%	0.141
LA- N (%)	(106) 75.7%	(33) 88.6%	(139) 78.1%	

LA+ with lymphoid aggregates; LA- without lymphoid aggregates.

Statistics: Age (independent t-test); Gender, LA (Fisher’s exact test).

The MDS group (n=140), included 34 patients (24.3%), in whom the BM biopsy contained lymphoid aggregates (MDS/LA+), and 106 patients without LA (MDS/LA-). Comparing the whole MDS group to the controls demonstrates a higher percentage of LA in MDS patients than in the controls: (24.3% vs 13.2%, respectively), although it failed to reach statistical significance (p=0.141, **Table 1**).

**Figure 1:**
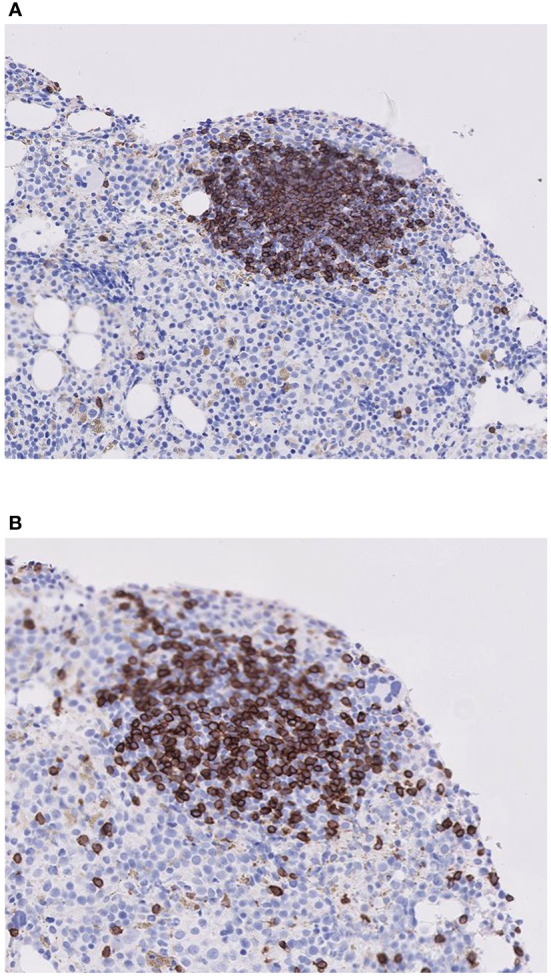
Histologic staining for B and T lymphocytes in a lymphoid aggregate for one representative patient. **(A)** Stain for B cells (CD20), **(B)** Stain for T cells (CD3). Note that the lymphoid aggregate contains a mixture of both B and T cells.

In 21 BM biopsy samples, staining had been performed for CD20 and CD3, markers of B cells and T cells, respectively. In 18 of them, both markers stained positive (**Figures 1A, B**), suggesting the likelihood of polyclonality of LA in these specimens and likely almost all of the rest as well.

In the analysis of the lymphoid markers on the CD34+ stem cells, we found that the percentage of positive markers (CD7 for T cells and CD19 for B cells) on these cells was similar for the MDS/LA+ and MDS/LA- groups (**Table 2**). Similarly, the percentage B and T cells among the lymphocytes and percentage of B and T cells among all BM cells was similar in both groups.

**Table 2:** Flow cytometry of B and T lymphoid markers on CD34+ stem cells, on lymphocytes, and in the total BM (MDS/LA+ vs MDS/LA- groups).

Marker	MDS/LA+ (%)	MDS/LA-(%)	P
Percentage of CD34+ Cells			
CD34+/CD7+	7.00%	6.35%	NS
CD34+/CD19+	8.20%	9.03%	NS
Percentage of Lymphocytes			
CD7+	73.82%	76.85%	NS
CD19+	13.65%	10.77%	NS
Percentage of Total BM			
Lymphocyte gate	17.03%	16.10%	NS
CD7+	12.90%	12.17%	NS
CD19+	2.15%	1.63%	NS

Statistics: t-test.

**Table 3** presents non-categorical continuous variables in the MDS patients with lymphoid aggregates (MDS/LA+) compared with the subgroup of MDS/LA-. The MDS/LA+ group is characterized by a trend toward younger age, lower values of Hb, MCV, WBC, ANC and platelet count, and a trend toward higher LDH level, BM cellularity, as well as a higher IPSS-R prognostic score. IPSS-R scores were available for 116 patients. A greater percentage of MDS/LA+ patients were categorized as higher risk (IPSS-R ≥ 3.5) than MDS/LA- patients (46% vs 32%). Despite the trends, none of these differences reached statistical significance.

**Table 3:** The non-categorical continuous variables in the MDS patients with lymphoid aggregates (MDS/LA+) compared with those of the subgroup MDS/LA-.

Parameter	MDS/LA+	MDS/LA-	P
N	34	106	
Age, years	72.2 ± 10.0	74.8 ± 11.0	0.143
Hb	9.8 [8.5-11.2]	10.2 [8.8-12.0]	0.192
MCV	94.8 ± 9.9	97.3 ± 10.1	0.218
WBC	3.7 [2.6-6.6]	6.0 [3.5-6.7]	0.065
ANC	1.8 [1.0-4.1]	2.6 [1.2-3.7]	0.166
Lymphocytes	1.2 [0.9-1.6]	1.5 [0.9-2.0]	0.094
Monocytes	0.5 [0.2-0.6]	0.4 [0.2-0.7]	0.987
PLT	102.5 [52.0-202.0]	118.0 [64.0-211.0]	0.451
LDH	472.0 [377.0-675.0]	417.0 [352.0-503.0]	0.183
BM Cellularity	50% [30%-75%]	40% [30%-70%]	0.397
*BM blast %	0% [0%-5%]	0% [0%-4%]	0.796
IPSS	0.5 [0.0-1.0]	0.5 [0.0-0.5]	0.19
IPSS-R	3.0 [1.5-5.0]	2.0 [1.5-4.0]	0.164
IPSS-R: LR n (%)	14 (54%)	61 (68%)	
IPSS-R: HR n (%)	12 (46%)	29 (32%)	

Hb – hemoglobin (g/dL); MCV, mean corpuscular volume (fL); WBC, white blood cells (x10^9^/L); ANC, absolute neutrophil count (x10^9^/L); PLT, platelets (x10^9^/L); LDH, lactate dehydrogenase (U/L); BM, bone marrow; IPSS, international prognostic scoring system; IPSS-R, IPSS revised; LR, lower risk (IPSS-R ≤ 3); HR, higher risk (IPSS-R ≥ 3.5).

*Median (not mean) of 0% BM blasts means that at least 50% of the patients had no blasts in the BM.

Age and MCV (normal distribution) are presented as mean ± SD; all others (non-normal distribution), as median [interquartile range, IQR].

Statistics: Age, MCV (independent t-test), All others (Mann Whitney test).

In **Table 4**, we describe comorbidities (categorical parameters), in the MDS/LA+ group compared with MDS/LA- patients. The MDS/LA+ and MDS/LA- patients had a similar incidence of cardiovascular diseases (CVD, 67.6% and 77.4% respectively). The incidence of cancer in the past was 26.5% and 14.3% respectively (p=0.102). Diabetes mellitus was significantly more common in the MDS/LA+ group: 38.2% vs 19.0% (p=0.022).

**Table 4:** Comorbidities in the MDS/LA+ patients compared with those in MDS/LA- patients.

	MDS/LA+	MDS/LA-	P
N	34	106	
CVD, N (%)	23 (67.6%)	82 (77.4%)	0.255
Cancer, N (%)	9 (26.5%)	15 (14.3%)	0.102
Diabetes, N (%)	13 (38.2%)	20 (19.0%)	0.022

CVD, cardiovascular diseases.

Statistics: Fisher’s exact test.

**Figure 2:**
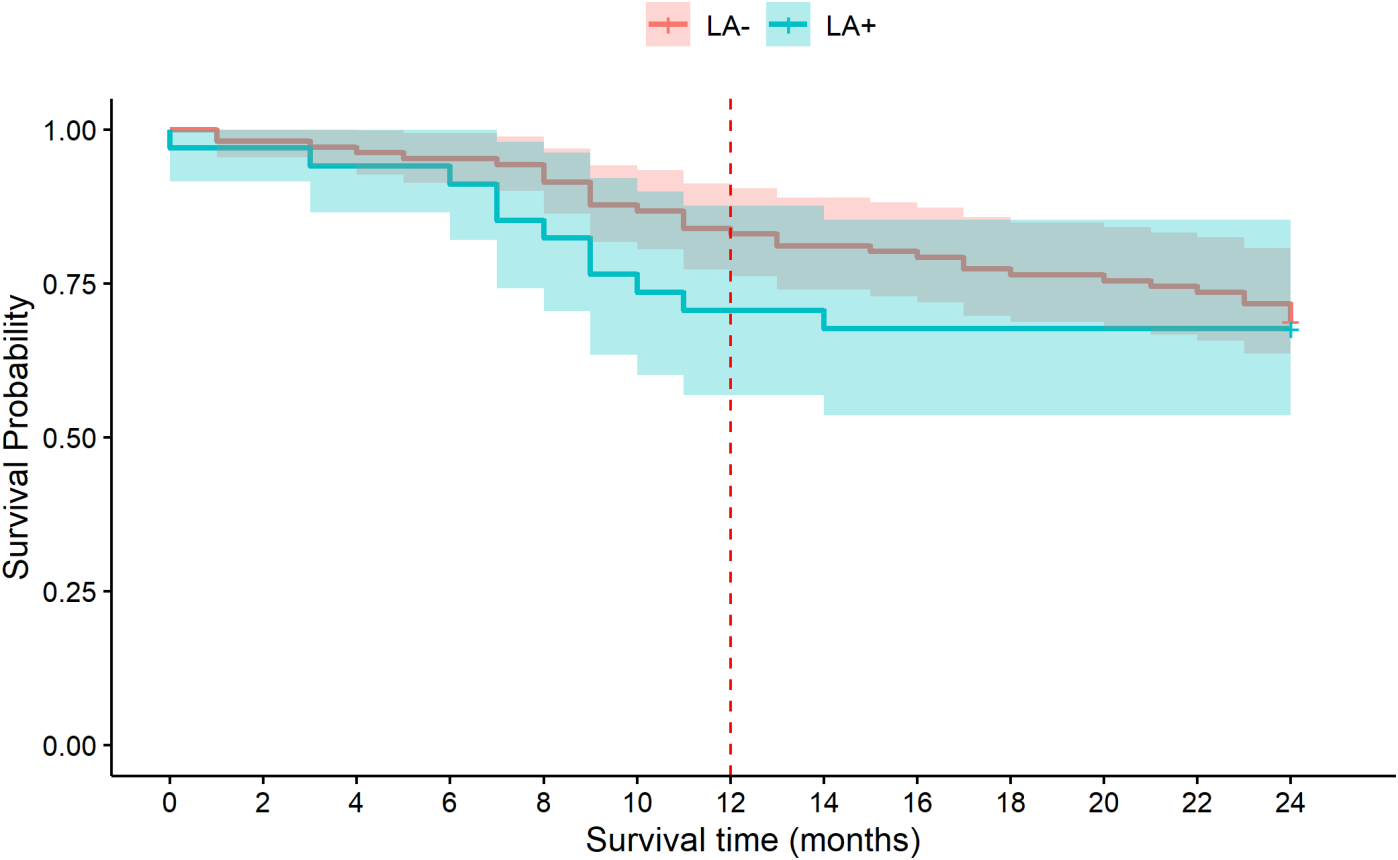
Kaplan-Meier (KM) survival curve of MDS patients with lymphoid aggregates (LA+, blue) and without lymphoid aggregates (LA-, orange) for the 24 months after diagnosis. The separation of the curves seen at 12 months (red dashed line) disappears at 24 months.

The survival of the two analyzed groups, MDS/LA+ and MDS/LA-, is demonstrated in **Table 5** and in **Figure 2**. The median survival after 24 months from diagnosis was 18.7 months and 20.55 for the LA+ and LA- groups, respectively, without statistical significance (p=0.692). The median survival is not reported at 12 months as more than 50% of the patients were alive at that point in time. The percentage of patients alive at 12 months was 70.6% (24/34) in the MDS/LA+ group and 83% (88/106) in the MDS/LA- groups (p=0.14, **Table 5**). This trend is reflected in the Kaplan-Meier survival curve (**Figure 2**, red dashed line). This difference disappears, however, at 24 months, where the percentage of patients alive at that time was similar in both groups: 67.6% (23/34) in the LA+ group and 68.9% (73/106) in the LA- group (P=1.0, **Table 5** and **Figure 2**).

**Table 5:** Survival of MDS patients with LA (MDS/LA+) and without LA (MDS/LA-).

N	MDS/LA+	MDS/LA-	P
	34	106	
Alive at 12m, N (%)	24 (70.6%)	88 (83%)	0.140
Alive at 24m, N (%)	23 (67.6%)	73 (68.9%)	1.000
Median 24m survival, months [95% CI]	18.7 [15.96, 21.45]	20.55 [19.32, 21.78]	0.692

Statistics: % Alive (Fisher’s exact test); Median survival (Mann Whitney test).

Finally, using the Cox proportional hazard model adjusted for the most relevant variables (age, gender, lymphocyte and WBC counts and diabetes), at 12 months lymphoid aggregates were associated with a hazard ratio (HR) greater than 2 with borderline statistical significance (p=0.055, **Table 6**, upper portion). At 24 months, this effect disappears (data not shown). The lower portion of **Table 6** shows the same model but with the inclusion of the IPSS-R score. As expected, the IPSS-R score is a significant predictor of mortality. In fact, within the first year 50% of the higher risk patients (IPSS-R ≥ 3.5) had died, while only 7.1% of the lower risk patients (IPSS-R ≤ 3) had died. The hazard ratio (HR) for LA in this model remained high, but with the broadening of the 95% confidence interval, it failed to reach statistical significance.

**Table 6:** Top portion.

	HR	[95% CI]	P
Age	1.000	[0.965-1.036]	0.996
Lymphoid Aggregates	2.283	[0.984-5.297]	0.055
Gender Female vs Male	0.601	[0.262-1.382]	0.231
Lymphocytes	0.815	[0.444-1.496]	0.509
WBC	1.055	[0.976-1.140]	0.178
Diabetes yes vs no	0.561	[0.212-1.483]	0.244
Inclusion of IPSS-R:			
Age	1.014	[0.968-1.062]	0.562
Lymphoid Aggregates	2.159	[0.810-5.572]	0.124
Gender Female vs Male	0.676	[0.263-1.741]	0.418
Lymphocytes	0.920	[0.525-1.611]	0.770
WBC	1.165	[1.058-1.281]	0.002
Diabetes yes vs no	0.528	[0.171-1.635]	0.268
IPSS-R	1.541	[1.288-1.844]	<0.001

Cox proportional hazard model of survival at 12 months for MDS patients with LA (MDS/LA+) and without LA (MDS/LA-), adjusted for age, gender, lymphocyte count and WBC count, and diabetes. Bottom portion. The same model with the inclusion of IPSS-R.

## Discussion

This study set out to assess lymphoid aggregates (LA) in the bone marrow biopsy (BMB) specimens of MDS patients. Our study confirms the relatively higher incidence of LA in BMB of MDS patients, compared with controls (24% vs 13%, although not statistically significant). Although specific clonality assays were not performed, the positivity of both CD3 and CD20, suggests that these LA are polyclonal. In MDS patients, the percentage of total lymphocytes, B cells and T cells (by flow cytometry) was similar, irrespective of the existence of lymphoid aggregates in the BM biopsy. MDS patients with LA (MDS/LA+) were younger, but had features of worse disease: lower values of all three hematologic cell lines, higher serum LDH, BM cellularity, as well as a higher IPSS-R score and a greater percentage in the higher risk (HR) category. However, despite this tendency of poor prognostic features in the MDS/LA+ group, none of these differences reached statistical significance. This may be due to the small number of analyzed patients in our study.

Scores such as IPSS and IPSS-R take into account several factors, including cytopenias, BM blasts, and basic cytogenetics, and are therefore strong predictors of mortality. Most recently, the IPSS-M score has been proposed, with the inclusion of more detailed molecular genetics (26).

Analyzing the survival, we found that there may be increased mortality for MDS/LA+ patients during the first 12 months from diagnosis as seen in **Table 6** and **Figure 2**. While this trend is borderline significant, in the model LA at 12 months had a hazard ratio greater than 2 (p=0.055). In other words, LA may potentially portend a worse prognosis at 12 months. This study leads one to ask further questions: whether the lymphoid aggregates play a role in the pathophysiology of MDS, or in patient survival, and why they might make no difference by 24 months.

If LA proves to be significant in continued studies with larger numbers of patients, then its incorporation into existing prognostic models (e.g. IPSS-R, IPSS-M) may provide added value.

On average, MDS/LA+ patients had approximately twice the incidence of past cancer and diabetes as compared to MDS/LA- patients, but only diabetes mellitus achieved statistical significance. The importance of this association remains to be clarified (27). There was a similar incidence of cardiovascular disease in both groups. A recent study demonstrated that the combination of comorbidities (reflected by increasing Charlson comorbidity index, CCI), malignancy, or renal disease was associated with worse overall survival in MDS patients (28). Our study might point to an association between comorbidities and LA in MDS patients, which would need to be studied further.

Do LA in the bone marrow of MDS patients have a role in the disease pathogenesis? The likely polyclonality of the aggregates themselves as well as the polyclonality seen in the lymphoid markers on the CD34+ cells, and in the BM in general, suggests a general immune response, and the relatively high incidence may suggest a biological role. It has been proposed that they represent an immunologic stimulation as a part of the pathobiology of MDS (20). Indeed, there is evidence about the crucial role of the immune system in the pathogenesis and the clinical manifestations of MDS (17, 19, 29–31). Along these lines, Silzle et al. have found that a low lymphocyte count is associated with a worse prognosis in otherwise low-risk MDS patients (32).

Our study has several limitations. The relatively small number of patients calls for caution in evaluating the results. It could also be the reason for the lack of statistical significance. The retrospective nature of this analysis is also a limitation. We assessed the BMB reports and while we had the analyses of CD20 and CD3 in a portion of the patients, and examined the flow cytometry in the cells, we performed no additional staining or molecular studies. It is also true that these data were not available for the BM of all patients. In this study, we refer to LA as a categorical parameter. We did not examine features of the aggregates like their number, size, and location within the BM. Also, because of the nature of this retrospective study, not all of the BMB specimens were of optimal quality.

The control group did not include truly healthy people. They were patients referred for a BMB for evaluation of a hematological problem, usually anemia, and their BMB was interpreted as normal. Ideally, one would like to use healthy individuals with normal BMB as controls, but they do not undergo such invasive procedures.

In addition, since the MDS patients were significantly older than the healthy controls, it might be argued that hematopoietic changes were related to clonal hematopoiesis with somatic mutations that have been found to exist in the elderly (33, 34). However, in the model (**Table 6**) age was not an important prognostic factor.

In summary, despite these limitations, we believe that this preliminary study teaches us several relevant lessons. LA appears to be present with a high enough incidence (24%) to warrant investigation. In our series, MDS patients with LA tended to have more baseline poor prognostic features, albeit without statistical significance. Of special interest is the association with diabetes, which should be studied further (27). While there are still more questions than answers, we hypothesize that this finding may reflect the role of the immune system in the pathogenesis of MDS. It is unclear at this time whether it has any relationship to the lymphocyte count or the other various immunologic and clinical manifestations commonly found in MDS. We present these data on a limited scale in order to stimulate further research on a larger scale. At this time, we are proceeding with this project to study much larger MDS patient and control cohorts, which could allow us to answer questions of LA significance as well as questions of basic pathophysiology.

## Data availability statement

The raw data supporting the conclusions of this article will be made available by the authors, without undue reservation.

## Ethics statement

The studies involving human participants were reviewed and approved by Helsinki Committee, Tel Aviv Sourasky Medical Center. Written informed consent for participation was not required for this study in accordance with the national legislation and the institutional requirements.

## Author contributions

RB collected the data, performed the initial analysis of the data, and wrote the first draft of the paper. JBE performed the assessment of bone marrow biopsies. CGS and SK performed and analyzed the flow cytometry of the BM aspirates. GS participated in data interpretation and writing the manuscript HO and MM designed the study, supervised the analysis, wrote the subsequent drafts of the manuscript. All authors contributed to the article and approved the submitted version.
